# Cranial Nerve III Palsy as the First Sign of Carcinomatous Meningitis From Non-Hodgkin’s Lymphoma

**DOI:** 10.7759/cureus.56277

**Published:** 2024-03-16

**Authors:** Gabriel Velez Oquendo, Sergio Alcantar, Sonu Gupta

**Affiliations:** 1 Internal Medicine, Northeast Georgia Medical Center Gainesville, Gainesville, USA; 2 Internal Medicine, Augusta University Medical College of Georgia, Augusta, USA

**Keywords:** non-hodgkin's lymphoma, cranial nerve iii palsy, oculomotor nerve (cn iii) palsy, leptomeningeal disease, carcinomatous meningitis

## Abstract

Carcinomatous meningitis (CM) is characterized by the multifocal dissemination of malignant cells into the cerebrospinal fluid (CSF), pia mater, and subarachnoid space. Involvement can occur in the advanced stage of malignancy, causing multifocal involvement and a wide array of symptoms. Diagnosis requires suspicions and a multimodal approach that includes imaging, lumbar puncture, and diagnostic laboratory evaluation. This case represents a female with a history of non-Hodgkin's lymphoma (NHL) and venous thromboembolism on chronic anticoagulation who presented due to acute encephalopathy, hallucinations, and right cranial nerve III palsy for 10 days before arrival. Computed tomography (CT) and angiography of the brain did not show any intracranial abnormalities. Subsequent magnetic resonance imaging (MRI) was without signs of infarction, hemorrhage, or abnormal enhancement, with the MRI of the orbits showing asymmetric linear enhancement anterior to the superior pons and midbrain on the right. Initial differential included a paraneoplastic syndrome, but there was no obvious evidence of pathological enhancement on MRI. Due to progressive bulbar symptoms, a lumbar puncture was performed with cerebrospinal fluid diagnostic workup with cytology showing monoclonal B-cell proliferation consistent with lymphoma. This case illustrates a rare but specific finding of CM as cranial nerve III palsy symptoms in this patient who did not have imaging findings that would reflect her symptoms on the initial MRI of the brain. Furthermore, diagnosing CM is complex and involves a combination of multiple diagnostic and treatment modalities. It is important to recognize the condition early to improve the patient's quality of life, prolong survival, and stabilize neurological deterioration.

## Introduction

Carcinomatous meningitis (CM), also known as leptomeningeal carcinomatosis, is described as the widespread extension of malignant cells to the subarachnoid space, pia mater, and cerebrospinal fluid (CSF) within the brain and spinal cord, potentially causing cranial nerve palsies [1-3]. The primary extracranial malignancies most frequently linked to CM are breast and lung cancer, melanoma, acute lymphoblastic leukemia (ALL), and non-Hodgkin's lymphoma (NHL) [2]. However, it is essential to note that almost any type of cancer has the potential to spread to the leptomeninges [2,3]. Clinical features are pleomorphic, such as headaches from elevated intracranial pressure (ICP), confusion, seizures, euphoria, and hallucinations [2]. The most prevalent symptoms are associated with cranial nerve dysfunction, such as diplopia, vision loss, sensory or motor loss in the trigeminal nerve, cochlear dysfunction, and optic neuropathy [4]. Consequently, a comprehensive history taking, and neurological examination determines whether there is focal or multifocal involvement. This leads a clinician to think there is leptomeningeal involvement and perform contrast-enhanced magnetic resonance imaging (MRI) of the brain, which is the imaging modality of choice, as well as CSF evaluation, while simultaneously ruling out alternative causes, like parenchymal disease, paraneoplastic syndromes, and infectious etiologies, as was done in this case [2-4]. This case describes a rare and unique presentation of CM as right-sided cranial nerve III palsy.

## Case presentation

Our case presents a 77-year-old female with a medical history of diffuse large B-cell NHL with recently completed treatment, deep venous thrombosis, and pulmonary embolism on chronic anticoagulation, hypothyroidism, hypertension, and generalized anxiety disorder who presented to the emergency department with complaints of acute encephalopathy associated with hallucinations, right-sided ptosis, progressive neuromuscular weakness, and fatigue for 10 days before arrival. She was hospitalized before presentation at another facility for the presenting symptoms, and she was found to have a hypertensive emergency and hyponatremia, but further evaluation including an MRI of the brain was without intracranial abnormalities. Despite persisting symptoms, the patient was discharged home, and the patient's husband decided to bring her to another facility for evaluation.

On presentation, the patient was found to have elevated blood pressure of 200/92 mmHg, heart rate of 78 beats/min, oxygen saturation at 96% on 2 L of supplementation via nasal cannula, and afebrile. Physical exam was notable for right-sided ptosis, restricted right-sided eye movement, anisocoria, confusion, dysarthria, and dysphagia to medications. Laboratory workup was remarkable for sodium level of 128 mmol/L (135-148 mmol/L), glucose of 110 mg/dL (65-99 mg/dL), and normal ammonia levels, urinalysis, and complete blood count. Initial chest X-ray was without acute cardiopulmonary abnormalities, and a CT of the brain without contrast did not show any intracranial processes. Consequently, the neurology service was consulted for evaluation of the patient and further recommendations.

After the initial workup, it was determined that the patient had hypotonic euvolemic hyponatremia secondary to a syndrome of inappropriate antidiuretic hormone (SIADH) with a serum osmolality of 272 mosm/kg (275-295 mosm/kg), a urine sodium level of 77 mmol/L (40-220 mmol/L), and urine osmolality of 483 mosm/KGH_2_O (500-800 mosm/KGH_2_O). Furthermore, the sedimentation rate was 34 mm (0-30 mm), the C-reactive protein was 3.90 mg/dL (0.00-0.60 mg/dL), and the thyroid panel, vitamin B12, folate, and vitamin D were normal. Computed tomography angiography (CTA) of the head and neck did not have evidence of large vessel occlusion or hemodynamically significant stenosis. MRI of the brain with and without contrast did not show abnormal enhancement, infarction, or hemorrhage, but it did show mild chronic microvascular ischemic changes. Given the absence of obvious evidence of pathological enhancement on imaging, it was decided to pursue a lumbar puncture for CSF evaluation (Table 1).

**Table 1:** Cerebrospinal fluid testing

CSF testing	Results	Reference range and units
White blood cell count	358/mm^3^	0-5/mm^3^
Red blood cell count	385/mm^3^	0-5/mm^3^
Protein	377 mg/dL	15.0-45.0 mg/dL
Glucose	12 mg/dL	40-80 mg/dL
Culture	No growth	No growth
Non-gynecologic cytology	Positive for CD-5 B cells	Negative
Multiple sclerosis IgG	25.1 mg/dL	0.0-6.0 mg/dL
Venereal disease research laboratory test (VDRL)	Non-reactive	Non-reactive
Angiotensin-converting enzyme	<0.4 U/L	0.0-0.5 U/L
Musk auto-antibody	0.00 nmol/L	0.00-0.02 nmol/L
Meningitis/encephalitis PCR	Negative	Negative
Anti-acetylcholine antibody	0.0 nmol/L	0.0-0.4 nmol/L
Ganglioside antibodies IgG & IgM	3 IV	0-50 IV
Paraneoplastic antibodies	Negative	Negative

**Figure 1:**
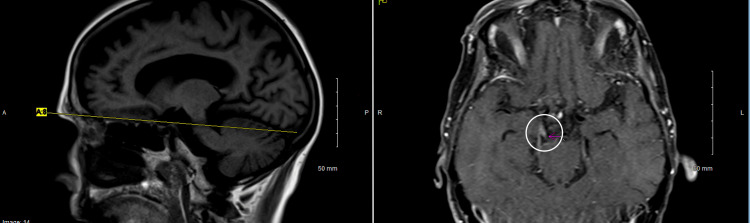
Axial post-contrast orbital MRI showing linear enhancement anterior to the superior pons and midbrain junction to the right of midline representing enhancement of the cisternal segment of the oculomotor nerve.

While the workup was pending, an MRI of the orbits was performed, and it was without abnormalities in the orbits but revealed asymmetric linear enhancement anterior to the superior pons and midbrain junction to the right of the midline representing enhancement of the cisternal segment of the oculomotor nerve (Figure 1). On reviewing the MRI for other abnormalities in proximity to the oculomotor nerve, nothing was found (Video 1). Finally, the CSF cytology testing had findings consistent with CD5-positive monoclonal B-cell proliferation, which was consistent with involvement by the previously diagnosed diffuse large B-cell NHL. In addition, the multiple sclerosis panel showed elevated immunoglobulin G in the CSF, but the test was altered by xanthochromia.

**Video 1:** Axial T1 thin FS view of the MRI of the oculomotor nerve without other cranial nerve abnormalities.

**Figure 2:**
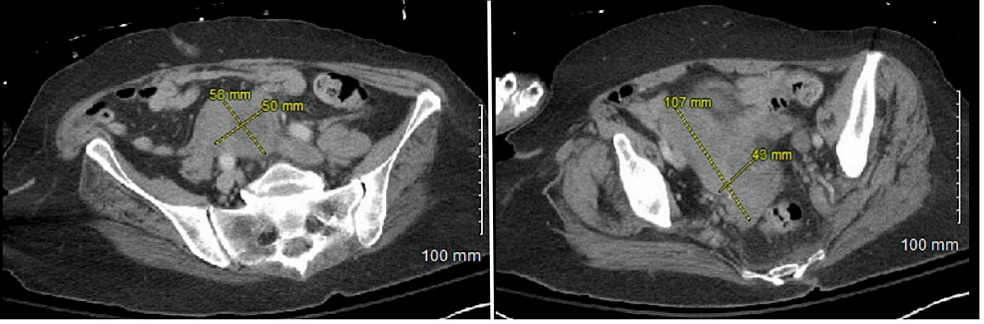
CT of the chest, abdomen, and pelvis showing findings compatible with worsening lymphatomatous disease, with right pelvic and mesenteric lymphadenopathy.

**Figure 3:**
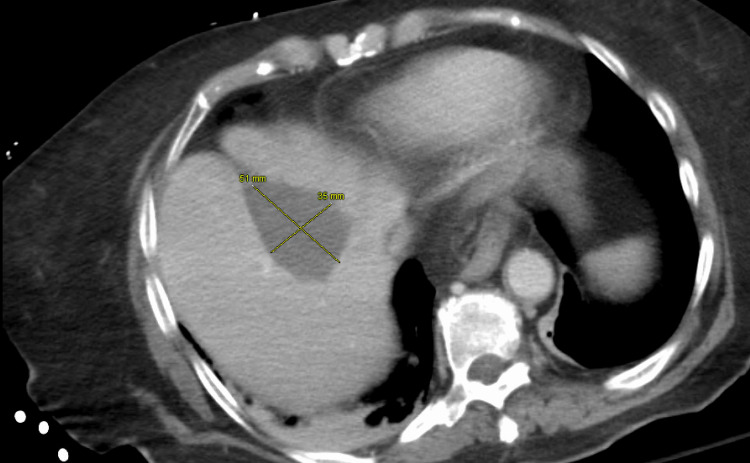
Persistent enhancing metastatic lesion in the liver.

To complete the patient’s work-up and evaluate the primary location of the patient’s malignancy a CT of the chest, the abdomen and pelvis showed findings compatible with worsening lymphomatous disease, with worsening right pelvic and mesenteric lymphadenopathy (Figure 2). In addition, there were persistent enhancing lesions in the liver (Figure 3).

Initially, the differential diagnosis for this patient's cranial nerve III palsy and associated symptoms included hyponatremia and hypertensive encephalopathy. After further evaluation, the possible etiologies evolved to include paraneoplastic syndrome affecting the mesencephalon, but there was no obvious evidence of pathological enhancement on MRI of the brain. Consequently, because the patient was having progressive bulbar symptoms including dysarthria, dysphagia to medications, and progressive neuromuscular weakness, atypical myasthenia gravis and Miller Fisher syndrome were also in the differential but were finally ruled out after the imaging only showed enhancement of the 3rd cranial nerve. It was concluded that the bulbar symptoms related to other cranial nerves could have been associated with microvascular changes. Furthermore, CSF evaluation was negative for those specific conditions, cytology was positive for monoclonal B-cells, and the team arrived at a final diagnosis of CM. Unfortunately, the patient's caretaker decided not to pursue further treatment, and the patient was discharged home with hospice.

## Discussion

CM or leptomeningeal disease (LMD) is described as the dissemination of cancer cells from a primary tumor site to the central nervous system (CNS) via hematogenous spread [2]. Diagnosis of LMD requires a high index of suspicion due to patients presenting with a wide array of clinical symptoms due to the simultaneous involvement of multiple locations throughout the neurological axis [5-6]. The incidence of LMD is around 5% in patients with metastatic cancer with the most common malignancies, including breast cancer, lung cancer, melanoma, and NHL [6-8].

The hematogenous spread usually occurs via the arachnoid vessels or direct extension from the brain parenchyma, with other possible routes, including retrograde invasion along peripheral or cranial nerves to the subarachnoid space [7]. Most episodes of CNS involvement by NHL occur in the setting of disease relapse [8-9]. Given the frequent multifocality, clinical presentation can be nonspecific with common clinical findings attributable to cranial and spinal nerve dysfunction, elevated intracranial pressure, and/or meningeal irritation [10]. The most common cranial nerves that are affected are VI, VII, and VIII, which lead to diplopia, facial weakness, and other associated symptoms, but not documented cranial nerve III palsy. Given the extensive presenting symptoms and frequently complex histories, consideration should be given to alternative diagnoses including meningitis, paraneoplastic syndromes, and autoimmune disorders. [10]

The diagnosis of LMD remains challenging with history taking paying specific attention to symptoms that are suggestive of multifocal involvement, coupled with a comprehensive neurological examination. MRI of the brain with contrast enhancement should be performed for patients suspected of having CM or other potential etiologies, such as metastases and/or alternative explanations for the specific cranial nerve neuropathy [8]. Lumbar puncture for CSF studies is not always necessary, but as in this case, it is essential when imaging is negative or nonspecific [8]. Despite the recommended intrathecal chemotherapy with high dose methotrexate >500 mg/m^2 ^and whole-brain radiation, the patient in this case and her family decided to avoid further treatment.

This case is unusual as it presents a patient with cranial nerve III palsy symptoms as the initial sole physical manifestation of underlying leptomeningeal disease from NHL, which is rare with a few documented cases of episodic oculomotor nerve palsy, and usually from breast malignancy [11-12]. The occurrence of oculomotor nerve palsy can be considered a potential differential for an underlying cause, as in this case with imaging that did not show contrast-enhancing lesions or associated surrounding edema [13-14]. Furthermore, there have been documented similar cases with cranial neuropathies associated with lymphoma in the CNS but with other associated clinical features, such as leukocytosis, lymphadenopathy, or "B-symptoms," that this patient did not have. In clinical cases, such as this scenario, establishing an accurate diagnosis is critical to guide the management of B-cell lymphoma.

## Conclusions

This case highlights the importance of comprehensive history taking and physical examination due to the diverse clinical features of the condition. Cranial nerve III palsy is a rare presentation of LMD and/or CM, and diagnosis requires a high index of suspicion due to the multifocal involvement of the CNS and the wide array of symptoms. In clinical cases, such as this scenario, initial imaging and evaluation were negative for intracranial abnormalities, for which a lumbar puncture and an orbital MRI were essential in establishing an accurate diagnosis. In LMD, it is critical to establish a diagnosis early in the course to guide management and prolong survival.
